# Virologic Response and Reinfection Following HCV Treatment among Hospitalized People Who Inject Drugs: Follow-Up Data from the OPPORTUNI-C Trial

**DOI:** 10.3390/v16060858

**Published:** 2024-05-27

**Authors:** Kristian Braathen Malme, Kathrine Stene-Johansen, Ingvild Klundby, Øystein Backe, Tarjei Foshaug, Maria Helseth Greve, Charlotte Meinich Pihl, Ane-Kristine Finbråten, Olav Dalgard, Håvard Midgard

**Affiliations:** 1 Department of Infectious Diseases, Akershus University Hospital, 1478 Lørenskog, Norway; olav.dalgard@medisin.uio.no; 2 Institute of Clinical Medicine, University of Oslo, 0371 Oslo, Norway; 3 Department of Virology, Norwegian Institute of Public Health, 0304 Oslo, Norway; kathrine.stene-johansen@fhi.no; 4 Department of Microbiology, Oslo University Hospital, 0424 Oslo, Norway; 5 Agency for Social and Welfare Services, 0182 Oslo, Norway; oystein.backe@vel.oslo.kommune.no (Ø.B.); tarjei.foshaug@vel.oslo.kommune.no (T.F.); 6 Foundation of Franciscan Aid, Nurses on Wheels, 0651 Oslo, Norway; m.greve@fransiskus.no; 7 Department of Medicine, Lovisenberg Diaconal Hospital, 0456 Oslo, Norway; charlottempihl@gmail.com; 8 Unger-Vetlesen Institute, Lovisenberg Diaconal Hospital, 0456 Oslo, Norway; ane.finbraten@gmail.com; 9 Department of Infectious Diseases, Oslo University Hospital, 0424 Oslo, Norway; 10 Department of Gastroenterology, Oslo University Hospital, 0424 Oslo, Norway; havardmi@gmail.com

**Keywords:** hepatitis C virus, elimination, sustained virological response, reinfection, people who inject drugs, direct-acting antivirals

## Abstract

Treatment of hepatitis C among people who inject drugs (PWID) may be complicated by loss to follow-up and reinfection. We aimed to evaluate sustained virologic response (SVR) and reinfection, and to validate complete pharmacy dispensation as a proxy for cure among PWID enrolled in a trial of opportunistic HCV treatment. Data were obtained by reviewing the electronic patient files and supplemented by outreach HCV RNA testing. Reinfection was defined based on clinical, behavioral, and virological data. Intention to treat SVR ≥ 4 within 2 years after enrolment was accomplished by 59 of 98 (60% [95% CI 50–70]) during intervention conditions (opportunistic treatment) and by 57 of 102 (56% [95% CI 46–66]) during control conditions (outpatient treatment). The time to end of treatment response (ETR) or SVR ≥ 4 was shorter among intervention participants (HR 1.55 [1.08–2.22]; *p* = 0.016). Of participants with complete dispensation, 132 of 145 (91%) achieved ETR or SVR > 4 (OR 12.7 [95% CI 4.3–37.8]; *p* < 0.001). Four cases of reinfection were identified (incidence 3.8/100 PY [95% CI 1.0–9.7]). Although SVR was similar, the time to virologic cure was shorter among intervention participants. Complete dispensation is a valid correlate for cure among individuals at risk of loss to follow-up. Reinfection following successful treatment remains a concern.

## 1. Introduction

Despite the declining prevalence of hepatitis C virus (HCV) infection worldwide, HCV transmission continues to occur,, with approximately 1.5 million new cases occurring annually [1]. Contrary to the WHO elimination targets [2], some countries are experiencing rising HCV incidence due to increasing injection drug use [3,4]. Globally, there are almost 15 million people who inject drugs (PWID), and more than 30% of those are living with chronic HCV infection [5]. Although direct-acting antiviral (DAA) HCV treatment is safe and effective among PWID [6,7,8,9] and successful models of care among PWID have been demonstrated in many settings [10,11,12,13,14,15], treatment uptake in this population remains suboptimal [16,17,18,19,20,21,22].

Comprising approximately 8900 individuals [23], the Norwegian PWID population has high coverage of opioid agonist therapy (OAT) and needle and syringe provision [24]. Nonetheless, HCV infection has been endemic among PWID, with a HCV RNA prevalence of 40–50% in the last few decades [25]. Facilitated by unrestricted DAA treatment since 2018 and low-threshold models of care, a marked decline in viremic prevalence has now been demonstrated [26,27]. 

HCV treatment among PWID may be complicated by low adherence [28], loss to follow-up [10,14,15,29], and reinfection following successful treatment [30,31,32,33]. Although reinfection rates are relatively low (3.7–4.9/100 PY) among PWID in Norway [10,34], alarmingly high rates (22.6–30.8/100 PY) have been reported from several countries, including the United Kingdom and Spain [29,35]. Thus, more data on virologic outcomes and reinfection rates are needed among the most marginalized PWID. HCV treatment trials have been challenging to conduct as loss of follow-up is common and documentation of sustained virologic response (SVR) can be difficult to obtain. More pragmatic outcome measures for HCV treatment are therefore needed.

OPPORTUNI-C was a cluster randomized trial among hospitalized PWID demonstrating the superiority of opportunistic HCV treatment in terms of treatment completion and time to treatment initiation [36]. The study also documented SVR, but within a very limited time frame. The aims of the present study were to assess SVR within 2 years, assess reinfection incidence, and to validate complete DAA dispensation from the pharmacy (i.e., treatment completion) as a proxy for SVR. 

## 2. Materials and Methods

### 2.1. Study Participants

OPPORTUNI-C (ClinicalTrials.gov NCT04220645) was a pragmatic, open-label, multicenter, stepped wedge cluster randomized trial conducted between 1 October 2019 and 31 December 2021 in departments of internal medicine, addiction medicine, and psychiatry at three hospitals in Oslo, Norway [36]. The study enrolled 200 participants, 98 during intervention conditions (immediate treatment) and 102 during control conditions (standard of care outpatient treatment). This follow-up study comprised all individuals enrolled in OPPORTUNI-C.

The study was approved by the Regional Committee for Medical Research Ethics in Norway on 3 March 2019 (reference number 2019-128) and was conducted according to the Declaration of Helsinki and the International Conference on Harmonization Good Clinical Practice guideline. Written, informed consent was obtained from all participants.

### 2.2. Data Collection

Baseline data, including sex, age, housing status, injecting drug use, OAT status, and stage of liver disease, was collected as part of the clinical trial [36]. Follow-up data were collected between 1 July 2022 and 8 August 2023. Data on DAA dispensations from the pharmacy were extracted retrospectively by reviewing the electronic patient files as previously described [36]. 

In the OPPORTUNI-C trial, treatment completion as assessed by the final DAA dispension from the pharmacy was the main outcome. In this study, the main outcome was SVR among study participants. Primary data on HCV RNA status was obtained by a retrospective review of the electronic hospital files and microbiology files from local and collaborating laboratories. In addition, efforts were made to reach treated participants with missing virologic data for follow-up HCV RNA testing. This included actively contacting participants by telephone, social media, or in person, and HCV RNA testing completed at the preferred site of the individual. This was carried out in collaboration with established low-threshold services for PWID in Oslo [10,37] and was conducted at existing low-threshold services, at the location/home of the participant, or at the outpatient department of one of the participating hospitals. HCV RNA testing was conducted either with venepuncture (using the cobas^®^ HCV Quantitative nucleic acid test on the cobas^®^ 6800/8800 Systems (Roche, Rotkreuz, Switzerland)) or with point-of-care testing using the Xpert^®^ HCV Viral Load Fingerstick test (Cepheid, Sunnyvale, CA, USA) (Supplementary Materials). A financial incentive of NOK 200 was offered for those with travel expenses for this visit.

All individuals with persistent viremia following treatment were approached for a semi-structured behavioral survey collecting data on self-reported DAA adherence, OAT status, and injecting risk behavior during the previous three months prior to the viremic sample. All viremic individuals were scheduled for retreatment as soon as possible.

### 2.3. Virologic Analyses

Whole genome sequencing (WGS) was performed on all stored baseline blood samples from OPPORTUNI-C and on recurrent viremic samples collected during follow-up. WGS was conducted at the Norwegian Institute of Public Health (NIPH) using the Illumina MiSeq-platform, and sequences from baseline and follow-up were compared to differentiate between relapse and reinfection (Supplementary Materials). If WGS was not possible, genotyping using a line probe assay was performed.

### 2.4. Outcomes

SVR was defined as undetectable HCV RNA at least four weeks after the estimated date of end of treatment (SVR ≥ 4) and within 2 years after enrolment in OPPORTUNI-C. Due to the natural dominance of control observations in the first part of the trial (as inherent in the stepped wedge cluster randomized design), a maximum of two years of observation time was used to limit discrepancies in observation time between control and intervention participants. Failure to achieve SVR was noted either if there was persistent viremia (i.e., treatment failure), if no samples were available for SVR assessment (i.e., loss to follow-up), if no DAAs were dispensed (i.e., lack of treatment), or if HCV RNA was undetectable but after 2 years of enrolment (i.e., SVR outside the time frame). End of treatment response (ETR) was defined as undetectable HCV RNA 0–4 weeks after the end of treatment (EOT).

All cases of persistent viremia were reviewed retrospectively, taking clinical (i.e., stage of liver disease, DAA regimen), virological (i.e., genotypes/sequence, timing of recurrence), and behavioral (i.e., injecting risk behaviors) characteristics into account [10]. Based on this assessment, reinfection was defined as recurrent viraemia following EOT in an individual where the risk of reinfection was considered higher than the risk of relapse or treatment failure due to non-adherence. Relapse was defined as viremia following EOT, where the risk of relapse was considered higher than the risk of reinfection and with self-reported adherence > 80%. Treatment failure due to non-adherence was defined as viremia following ETR, where the risk of treatment failure was considered higher than the risk of reinfection and with self-reported adherence <80%.

### 2.5. Statistical Analysis

Data are reported as N (%), median (Interquartile range (IQR)) or mean (standard deviation (SD)). SVR was analyzed according to intention-to-treat (ITT) among all enrolled participants. SVR during intervention and control conditions is reported as proportions and risk differences with 95% exact confidence intervals (CI).

Time to ETR or SVR was evaluated using Kaplan–Meier analysis and Cox regression reporting hazard ratios (HR), with cluster as a shared frailty factor [38]. The time of risk was from the date of enrolment until the date of SVR, death, or 2 years after enrolment, whatever came first. The proportional hazard′s assumption was tested using Schoenfeld residuals and log–log transformation of the failure function.

All individuals with available HCV RNA samples following EOT were eligible for reinfection analysis. Reinfection incidence rates were calculated assuming a Poisson distribution, and the time of risk was calculated from EOT until the last undetectable HCV RNA for individuals without reinfection or until the first detectable HCV RNA for individuals with reinfection.

A sensitivity analysis was performed, comparing SVR results within 2 years to SVR results for the entire observation period.

All analyses were performed in STATA v.17.0 (College Station, TX, USA).

## 3. Results

### 3.1. Study Participants

**Figure 1:**
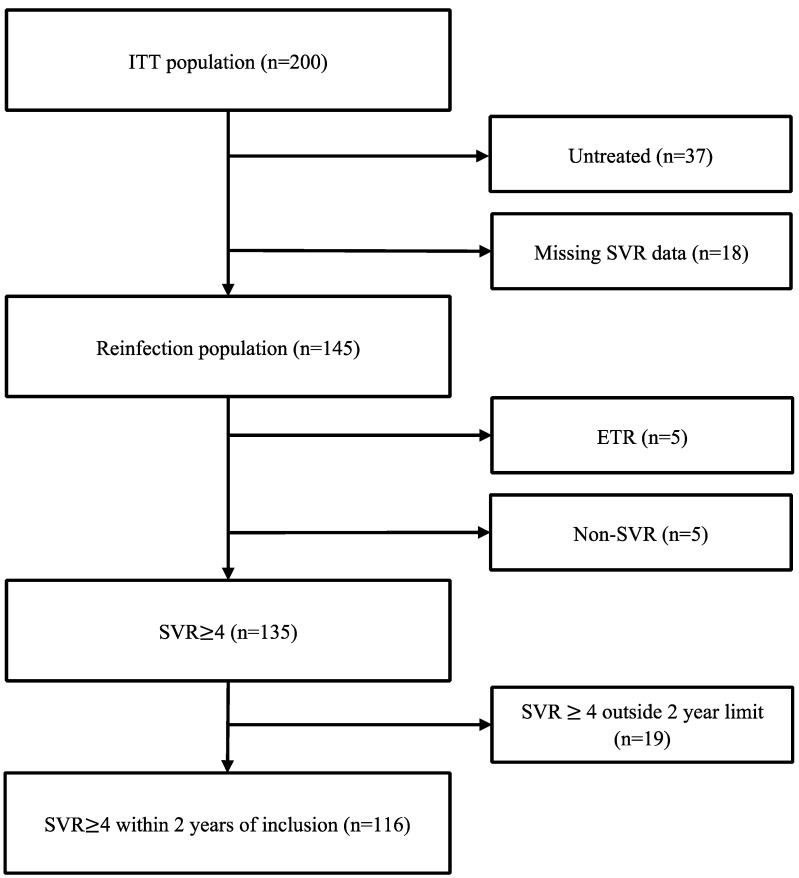
Flow chart illustrating SVR data for study participants.

The ITT population comprised all 200 individuals enrolled in OPPORTUNI-C (Figure 1).

The mean age was 47.4 years, 73% were male, 46% received OAT, and 61% reported recent injecting drug use (Table 1). Within 28 months following enrolment, 163 of 200 individuals (82%) had initiated HCV treatment (87/98 (89%) during intervention conditions, 76/102 (75%) during control conditions), of whom 4 were treated after data lock of the initial trial. HCV RNA follow-up samples were available for 166 of 200 individuals; 139 were collected based on retrospective review, and 27 were collected by outreach using venepuncture (*n* = 23) or point-of-care testing using the Xpert^®^ HCV Viral Load Fingerstick test (*n* = 4). 

### 3.2. Virologic Response

SVR ≥ 4 within 2 years of enrolment (Table 2) was documented in 59 of 98 (60% [95% CI 50–70]) during intervention conditions and in 57 of 102 (56% [95% CI 46–66]) during control conditions (risk difference 4% [95% CI −9–18]). SVR ≥ 4 was numerically higher among females and among those with recent injecting drug use and unstable housing, particularly during intervention conditions (Table 2). A sensitivity analysis comparing SVR ≥ 4 within 2 years of enrollment to SVR ≥ 4 not limited to two years is shown in Supplementary Tables S1 and S2. 

Reasons for failure to achieve SVR ≥ 4 (Supplementary Table S3) were most frequently explained by missing SVR data (*n* = 20) during intervention conditions and by lack of treatment (*n* = 26) during control conditions, while treatment failure was rare during both conditions (*n* = 5). Death during follow-up (*n* = 24) explained missing SVR data in 6 individuals and lack of treatment in 12 individuals, while 6 individuals died after achieving SVR.

**Figure 2:**
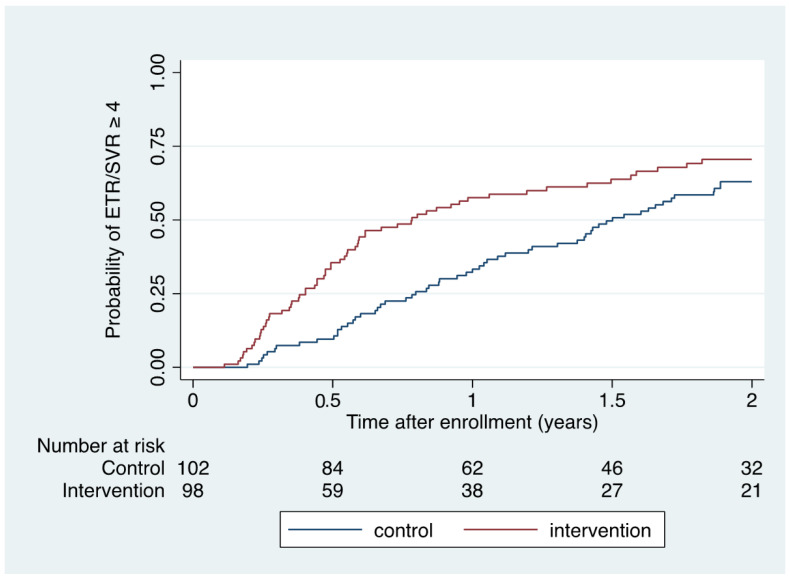
Kaplan–Meier estimate of time to ETR/SVR ≥ 4 according to intervention and control conditions.

When also considering those who achieved ETR but had missing SVR data (*n* = 5), ETR or SVR ≥ 4 was accomplished in 63 of 98 (64% [95% CI 54–74]) during intervention conditions and in 58 of 102 (57% [95% CI 47–67]) during control conditions (risk difference 7% [95% CI -6–21]). Kaplan–Meier (Figure 2) and Cox regression analysis demonstrated a significantly shorter time to ETR or SVR ≥ 4 within 2 years of enrolment among intervention participants compared to control participants (HR 1.55 [1.08–2.22]; *p* = 0.016; log rank *p* = 0.015).

### 3.3. Validation of Complete Dispensation

Among 145 participants who were treated with either intervention or control conditions and dispensed the final DAA package from the pharmacy (i.e., treatment completion), SVR ≥ 4 could be documented in 127 (88%) with an additional 5 (3%) achieving ETR, 3 (2%) had treatment failure, and 10 (7%) were lost-to follow-up (Table 3). Among 18 participants who were only dispensed the first DAA package (i.e., treatment discontinuation), 8 (44%) achieved SVR ≥ 4, none achieved ETR, while 2 (12%) had treatment failure and 8 (44%) were lost to follow-up. 

Among the 163 individuals who initiated treatment, a complete DAA dispensation from the pharmacy was associated with more than 12-fold increased odds of achieving ETR or SVR > 4 compared to an incomplete dispensation (OR 12.7 [95% CI 4.3–37.8]; *p* < 0.001).

### 3.4. Reinfection

A total of 145 individuals were eligible for reinfection analysis, contributing a total of 106.1 PY of observation. At enrolment, the mean age was 46.8 years, 73% were male, 47% received OAT, and 62% had recently injected (past 3 months).

**Figure 3:**
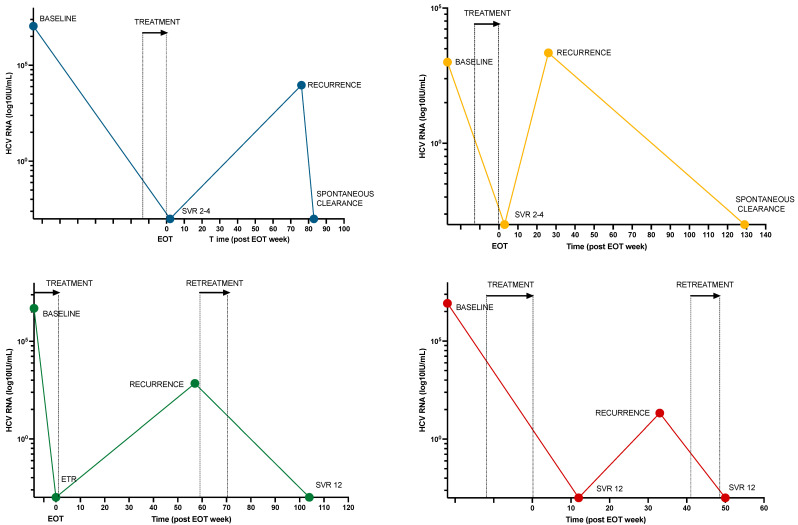
HCV RNA viral load over time for 4 participants with HCV reinfection. Colored dots represent HCV RNA samples at various times for each participant. Treatment and retreatment times are marked with an arrow and stipulated lines. Abbreviations: EOT, end of treatment; SVR, sustained viral response; ETR, end-of-treatment response.

A total of 17 individuals (12%) had persistent or recurrent viremia after treatment, of whom 6 had recurrent viremia following ETR (*n* = 3) or SVR4-12 (*n* = 3). WGS was performed on both baseline and recurrent samples in 5 cases. Based on a review of clinical, virological, and behavioral characteristics, 4 cases (3%) of reinfection, 1 case (1%) of relapse, and 12 cases (8%) of treatment failure due to low adherence were identified (Figure 3 and Table 4). 

The incidence of reinfection was 3.8/100 PY (95% CI 1.0–9.7) overall and 4.1/100 PY (95% CI 0.8–11.9) among those with recent injection drug use at enrolment (Supplementary Table S4). The median time from the end of treatment to reinfection was 0.83 years (IQR 0.56–1.25), with all four cases detected during the first year of follow-up. 

All four reinfections occurred in men younger than 50 years. Analysis of reinfection rates showed a signal that rates were lower among intervention participants (2.4/100 PY [95% CI 0.1–13.6]) compared to control participants (4.8/100PY [95% CI 1.0–9.7]), although it did not reach statistical significance. Kaplan–Meier analysis of time to reinfection stratified by key variables is shown in Supplementary Figure S1.

### 3.5. Retreatment

Retreatment was scheduled for all 17 individuals with persistent viremia. Of those, 10 achieved SVR ≥ 4 (of whom 3 outside the 2-year limit), one had virologic failure, and four were lost to follow-up without any retreatment (Supplementary Table S4). Of the four reinfection cases, two were successfully retreated, while two resolved spontaneously (Figure 3). The median time from viremic sample to retreatment was 3 weeks (IQR 1.9–19.7 weeks). By the time of study completion, five individuals were still living with a chronic HCV infection. No second reinfections were observed. 

## 4. Discussion

This study evaluated SVR and reinfection among participants enrolled in a cluster randomized trial of immediate HCV treatment in hospitalized PWID (OPPORTUNI-C). Although SVR after 2 years was similar in the two groups, the time to virologic cure was shorter among intervention participants. Reinfection incidence was moderate and in line with findings from previous studies in Norway. Importantly, high SVR among participants with complete DAA dispensation validates a new pragmatic correlate for cure that could be adopted in other low-threshold settings and aid HCV elimination efforts among PWID. 

SVR was lower than in most previous HCV treatment studies among PWID [6,7,8,11,13]. This could be explained by the pragmatic study design, which facilitated broader recruitment of more marginalized individuals than in conventional clinical trials. In particular, hospitalization due to complications of injection drug use could represent an additional vulnerability compared to non-hospitalized PWID. Notably, SVR rates were highest among younger individuals, females, those with unstable housing, and those with recent injecting drug use. This could be explained by increased testing activity facilitated by collaborating low-threshold services during data collection. This finding adds to previous literature supporting equally high treatment efficacy in highly marginalized groups [8,10,15,29].

Although SVR was similar during both conditions, the primary reason for non-SVR during intervention conditions was missing data due to loss to follow-up, while lack of treatment was the most common reason during control conditions. Thus, the similar SVR rates could be explained by the intervention reaching more marginalized individuals at higher risk of loss to follow-up rather than the intervention being ineffective. Furthermore, because high SVR rates can be achieved despite suboptimal adherence [39] or treatment interruption [40], SVR is probably underestimated among intervention participants.

Although treatment uptake was slower during control conditions, we observed that many control participants ultimately received treatment in low-threshold HCV programs in the City of Oslo [10,37]. As expected from the naturalistic features of the trial, this may have ‘diluted’ the intervention and contributed to an underestimation of the intervention effect compared to settings without access to similar low-threshold care. Nevertheless, the significant loss to follow up among intervention participants highlights the need for additional services to keep marginalized individuals engaged in care.

A complete DAA dispensation was associated with 12-fold increased odds of achieving ETR or SVR compared to those with incomplete dispensation. This suggests that complete dispensation from the pharmacy could be used as a proxy for cure in populations with a similar HCV prevalence. This is a novel finding, and to our knowledge, this is the first study to validate such a pragmatic correlate for cure. Because HCV RNA samples to assess SVR can be difficult to obtain in marginalized individuals at risk of loss to follow-up, shorter and more pragmatic approaches tailored to PWID are needed to aid elimination efforts in this population. 

The incidence of reinfection was similar to previous studies from Norway [10,34] and in line with most international studies among PWID [30,32,33]. Reinfection exclusively occurred in males below 50 years old, and rates were also higher in those with unstable housing and recent injecting drug use. As HCV elimination efforts have progressed and harm reduction coverage has increased, HCV prevalence in Norway has declined dramatically during the last decade [26,27]. Therefore, it is both surprising and concerning that the incidence of reinfection has remained unchanged. Although one possible explanation is the enrolment of more marginalized individuals than in previous studies, the results still suggest that existing harm reduction services may be insufficient to reduce risk among younger males. Although numerically lower rates among intervention participants could be due to coincidence, it raises the hypothesis of a protective effect of being engaged in such a proactive model of HCV care. 

Few previous studies have documented the retreatment of reinfection [10]. Because detection and retreatment of reinfections are key to HCV elimination, it is reassuring that all cases were successfully cleared and that no second reinfections were observed. Although most cases with persistent viremia for other reasons were also successfully retreated, five individuals were still living with chronic HCV infection at study completion. Thus, despite high harm reduction coverage and a declining HCV prevalence, we still need improvements in the delivery of care. 

Collectively, the findings add weight to the conclusions from OPPORTUNI-C [36], corroborating the feasibility and efficacy of an opportunistic treatment model among hospitalized PWID. This is the first study to validate complete DAA dispensation as a correlate for SVR. Given the availability of similar pharmacy-based data and similar viremic prevalence, this could meet the need for a shorter and more pragmatic proxy for cure that could be adopted by global partners in HCV elimination. Finally, the persistent reinfection risk is a reminder to all stakeholders that reinfection remains a challenge despite high harm reduction coverage and considerable reductions in HCV prevalence.

Key strengths of the study relate to the pragmatic features of OPPORTUNI-C, which aimed to generate optimal conditions for generalizability. This includes (1) broad and relatively unbiased recruitment of marginalized PWID; (2) an ITT principle for SVR analysis illustrating a ‘real-world’ and naturalistic HCV micro-elimination experience; and (3) the combined use of registry-based and prospective data collection to reduce the impact of loss to follow-up. Notably, the use of point-of-care testing in marginalized populations may be key to achieving HCV elimination among PWID [12]. Lastly, the combination of clinical, behavioral, and virological data with whole genome sequencing has contributed to a relatively robust differentiation between reinfection, relapse, and virologic failure. 

The study had several weaknesses. First, prospective HCV RNA testing was only scheduled for individuals who had initiated treatment during the time frame of the original trial. Because treatment initiation was more frequent during intervention conditions, this may have favored higher SVR rates among intervention participants. Second, using ETR or SVR4 as a surrogate of cure deviates from the traditional definition of virologic cure (i.e., SVR12). Although this may hamper direct comparison with the broader HCV literature, it is probably a more feasible endpoint among individuals at risk of loss to follow-up. Third, reinfection estimates and analysis of associated factors are limited by the few reinfection cases, small sample size, and relatively short individual follow-up time. Also, due to variable HCV RNA testing intervals, cases of reinfection with spontaneous clearance may have been missed. It is also possible that individuals with a higher risk of reinfection to a greater degree were lost to follow-up. Both factors may have led to an underestimation of the incidence of reinfection. Finally, as whole genome sequencing was accomplished in only a limited number of samples with recurrent or persistent viremia, misclassification may have occurred.

## 5. Conclusions

Although SVR within 2 years was similar, the time to virologic response was shorter among intervention participants, corroborating the efficacy of the opportunistic treatment model previously documented. Complete treatment dispensation could meet the need for shorter and more pragmatic correlates for cure among marginalized individuals at risk of loss to follow-up. Despite declining viremic prevalence among PWID, reinfection remains a challenge that needs to be addressed.

## Figures and Tables

**Table 1:** Baseline characteristics summarized by Total and Intervention Conditions.

Variable	Total (*n* = 200)	Intervention (*n* = 98)	Control (*n* = 102)
**Age, mean (SD)**	47.4 (12.7)	48.0 (13.0)	46.8 (12.5)
**Age groups**			
20–34	39 (20)	20 (20)	19 (19)
35–49	73 (37)	28 (29)	45 (44)
50–80	88 (43)	50 (51)	38 (37)
**Sex**			
Male	145 (72)	69 (70)	76 (75)
Female	55 (28)	29 (30)	26 (25)
**Housing status**			
Stable	124 (62)	64 (65)	60 (59)
Unstable	76 (38)	34 (35)	42 (41)
**History of injecting drug use**			
Yes	183 (92)	86 (88)	97 (95)
No	17 (8)	12 (12)	5 (5)
**Recent (past 3 months) injecting drug use**			
Yes	121 (60)	58 (59)	63 (62)
No	79 (40)	40 (41)	39 (38)
**Preferred injected drug †**			
Heroin	114 (64)	54 (64)	60 (65)
Amphetamines	51 (29)	25 (29)	26 (28)
Other/mixed	12 (7)	6 (7)	6 (7)
**Current opioid agonist therapy**			
Yes	90 (45)	38 (39)	52 (51)
No	110 (55)	60 (61)	50 (49)
**Liver cirrhosis**			
Mild or no liver fibrosis	156 (80)	76 (78)	80 (82)
Liver cirrhosis	40 (20)	22 (22)	18 (18)
**Charlson comorbidity index**			
0–1	101 (51)	41 (42)	60 (59)
2–3	43 (21)	30 (31)	13 (13)
≥4	56 (28)	27 (27)	29 (28)
**Discipline**			
Internal medicine	107 (54)	57 (58)	50 (49)
Addiction	65 (33)	25 (26)	40 (39)
Psychiatry	28 (1)	16 (16)	12 (12)

Numbers are shown as n (%) unless otherwise indicated. Missing values are excluded from percentages. Abbreviations: SD, standard deviation. † Among those with a history of injecting drug use; missing data for 7 participants (2 intervention, 5 control). Based on liver stiffness measurements in 86, FIB-4 (fibrosis-4) index in 107, and imaging in 3 participants: missing data for 4 control participants.

**Table 2:** Intention to treat SVR ≥ 4 within 2 years of enrolment in subgroups according to intervention and control conditions.

Variable	Total		Intervention		Control	
	*n*/N	% (95% CI)	*n*/N	% (95% CI)	*n*/N	% (95% CI)
**Overall**	116/200	58 (51–65)	59/98	60 (50–70)	57/102	56 (46–66)
**Age groups**						
20–34	23/39	59 (42–74)	10/20	50 (27–73)	13/19	68 (43–87)
35–49	46/73	57 (51–74)	19/28	68 (48–84)	27/45	60 (44–74)
50–80	47/88	53 (43–64)	30/50	60 (45–74)	17/38	44 (28–62)
**Sex**						
Male	81/145	56 (47–64)	39/69	57 (44–68)	42/76	55 (43–67)
Female	35/55	64 (50–76)	20/29	69 (49–85)	15/26	58 (37–77)
**Housing status**						
Stable	67/124	54 (45–63)	32/64	50 (37–63)	35/60	58 (45–71)
Unstable	49/76	65 (53–75)	27/34	79 (62–91)	22/42	52 (36–68)
**Recent (past 3 months) injecting drug use**						
Yes	75/121	62 (53–71)	37/58	64 (50–76)	38/63	60 (47–72)
No	41/79	52 (40–63)	22/40	55 (39–70)	19/39	49 (32–65)
**Preferred injected drug †**						
Heroin	66/114	58 (48–67)	32/54	59 (45–72)	34/60	64 (43–69)
Amphetamines	26/51	50 (37–65)	14/25	56 (35–76)	12/26	46 (27–67)
Other/mixed	8/12	67 (35–90)	4/6	67 (22–96)	4/6	67 (22–96)
**Current opioid agonist therapy**						
Yes	56/90	62 (51–72)	27/38	74 (54–85)	29/52	56 (41–70)
No	60/110	55 (45–64)	32/60	53 (40–66)	28/50	56 (41–70)
**Liver cirrhosis**						
Mild or no liver fibrosis	93/156	60 (52–67)	46/76	61 (49–72)	47/80	59 (47–70)
Liver cirrhosis	22/40	55 (39–71)	13/22	59 (36–79)	9/18	50 (26–74)
**Charlson comorbidity index**						
0–1	63/101	62 (52–72)	26/41	63 (47–77)	37/60	62 (48–74)
2–3	26/43	61 (44–75)	20/30	66.7 (47–83)	6/13	46 (19–75)
≥4	27/56	48 (35–62)	13/27	48.1 (29–68)	14/29	48 (29–68)
**Discipline**						
Internal medicine	53/107	50 (40–59)	34/57	59 (46–72)	19/50	38 (25–53)
Addiction	43/65	66 (53–77)	16/25	64 (43–82)	27/40	68 (51–81)
Psychiatry	20/28	71.4 (51.3–86.8)	9/16	56.3 (29.9–80.2)	11/12	91.7 (61.5–99.7)

† Among those with a history of injecting drug use; missing data for 7 participants (2 intervention, 5 control). Based on liver stiffness measurements in 86, FIB-4 (fibrosis-4) index in 107, and imaging in 3 participants, missing data for 4 control participants.

**Table 3:** Virologic outcomes among participants who initiated treatment according to treatment completion and treatment discontinuation as assessed by dispensation data from the pharmacy.

	Total (*n* = 163)	Treatment Completion (*n* = 145)	Treatment Discontinued (*n* = 18)
SVR ≥ 4	135 (83)	127 (88)	8 (44)
ETR	5 (3)	5 (3)	0 (0)
Treatment failure	5 (3)	3 (2)	2 (12)
Missing SVR data	18 (11)	10 (7)	8 (44)

Numbers are shown as *n* (%). Abbreviations: SVR, Sustained Virologic Response; ETR, End of treatment response.

**Table 4:** Characteristics of 17 individuals with viremia following initiation of hepatitis C treatment.

ID	Sex and Age	Cirrhosis ^†^	Treatment Regimen	Treatment Adherence	Sequencing Baseline	Sequencing Recurrent	GenotypeShift	Time to Event ^§^	Recent Injecting	Recent Sharing	Recent OAT	Diagnosis	Time to Retreatment ^$^	Retreatment Regimen	Outcome
1	M, 43	No	SOF/LDV	100%	Yes	No **	1a–?	76	Yes	Yes	Yes	Reinfection	N/A	Untreated	Spontaneous clearance
2	M, 37	No	SOF/VEL	-	No *	No **	1a–?	26	Yes	-	Yes	Reinfection	N/A	Untreated	Spontaneous clearance
3	M, 22	No	SOF/VEL	98%	Yes	No *	3a–3a	54	Yes	Yes	Yes	Reinfection	2	SOF/VEL/VOX	SVR ≥ 12
4	M, 48	No	SOF/VEL	-	Yes	No *	3a–?	33	-	-	-	Reinfection	1	GLE/PIB	SVR 4–12
5	F, 24	No	SOF/LDV	40%	No*	No *	1a–1a	23	No	No	Yes	Non-adherence	110	SOF/LDV	SVR ≥ 12
6	F, 44	No	GLE/PIB	0%	Yes	No **	3a–?	64	Yes	-	Yes	Non-adherence	61	GLE/PIB	SVR 4–12
7	M, 66	No	SOF/VEL	20%	Yes	Yes	2b–2b	80	Yes	Yes	Yes	Non-adherence	N/A	Untreated	Non-SVR
8	M, 47	No	SOF/VEL	30%	No *	No *	3a–3a	18	No	No	No	Non-adherence	13	SOF/VEL/VOX	SVR ≥ 12
9	M, 34	No	GLE/PIB	60%	Yes	No *	3a–3a	23	Yes	No	Yes	Non-adherence	1	GLE/PIB	SVR ≥ 12
10	F, 67	No	GZR/EBR	0%	Yes	No **	1a–?	58	No	No	No	Non-adherence	3	SOF/VEL	Non-SVR
11	M, 52	No	GLE/PIB	50%	No**	No **	?–1a	44	-	-	Yes	Non-adherence	N/A	Untreated	Non-SVR
12	F, 42	No	GLE/PIB	0%	Yes	Yes	3a–3a	43	No	No	No	Non-adherence	3	GLE/PIB	SVR ≥ 12
13	M, 49	No	SOF/VEL	10%	Yes	No **	1a–1a	1	-	-		Non-adherence	2	GZR/EBR	SVR 4–12
14	M, 52	No	SOF/VEL	50%	Yes	Yes	3a–3a	17	No	No	Yes	Non-adherence	48	SOF/VEL	SVR ≥ 12
15	M, 49	No	SOF/VEL	25%	Yes	No **	4d–?	3	No	No	No	Non-adherence	N/A	Untreated	Non-SVR
16	M, 69	Yes	SOF/VEL + R	100%	Yes	Yes	3a–3a	39	No	No	No	Relapse	20	SOF/VEL/VOX+ R after liver Tx	SVR ≥ 12
17	M, 44	Yes	SOF/VEL + R	50%	Yes	Yes	3a–3a	5	No	No	Yes	Non-adherence	N/A	Untreated	Non-SVR

Abbreviations: IQR, interquartile range; SOF/VEL, sofosbuvir/velpatasvir; GLE/PIB, glecaprevir/pibrentasvir; SOF/LDV, sofosbuvir/ledipasvir; GZR/EBR, grazoprevir/elbasvir; SOF/VEL/VOX, sofosbuvir/velpatasvir/voxilaprevir; R, Ribavirin N/A= not applicable. * Sequencing not performed because HCV RNA viral load < 50,000. ** Sequencing not performed because of other reasons, such as failure during analysis, missing sample. ^§^ Estimated time (weeks) from end of treatment to the event. ^$^ Estimated time (weeks) from detection of the event to retreatment initiation. ^†^ Cirrhosis (Y/N) based on liver stiffness measurements in 10, FIB-4 index in 7 participants.

## Data Availability

The data underlying this article are not available for legal, privacy, and ethical reasons.

## Supplementary Materials

The following supporting information can be downloaded at: https://www.mdpi.com/article/10.3390/v16060858/s1, Supplementary methods, Supplementary results, Table S1: Baseline characteristics for the ITT population, summarized by total, SVR ≥ 4 within 2 years of inclusion, and SVR ≥ 4 for total observation time; Table S2: Intention to treat SVR ≥ 4 within 2 years of enrolment vs. SVR ≥ 4 for total observation time in subgroups according to intervention and control conditions; Table S3: Virologic outcomes and reasons for failure to accomplish SVR ≥ 4 within 2 years of enrollment according to intervention and control conditions. Figure S1: Kaplan-Meier estimates of time to reinfection according to key variables.
