# Supplementary material for: Virologic Response and Reinfection Following HCV Treatment among Hospitalized People Who Inject Drugs: Follow-Up Data from the OPPORTUNI-C Trial
Source: Viruses. 2024 May 27;16(6):858. doi: 10.3390/v16060858 (PMC11209464; doi:10.3390/v16060858)

Supplementary Materials

Table of contents:

1. **Supplementary methods**
2. **Supplementary results**
3. **Supplementary Table S1.** Baseline characteristics for the ITT population, summarized by total, SVR≥ 4 within 2 years of inclusion, and SVR≥ 4 for total observation time.
4. **Supplementary Table S2.** Intention to treat SVR≥ 4 within 2 years of enrolment vs. SVR≥ 4 for total observation time in subgroups according to intervention and control conditions.
5. **Supplementary Table S3.** Virologic outcomes and reasons for failure to accomplish SVR≥ 4 within 2 years of enrollment according to intervention and control conditions
6. **Supplementary Table S4.** Subgroup incidence rates of reinfection
7. **Supplementary Figure S1.** Kaplan-Meier estimates of time to reinfection according to key variables.

1. Supplementary methods

1.1 Supplementary text regarding HCV RNA testing

HCV RNA testing was performed prospectively for 27 participants. For 6 of these individuals, HCV RNA testing was done by venepuncture at the nearest hospital. For 17 individuals, testing was done by an outreach visit of a nurse from the Hepatitis C clinic of Oslo Municipality, using venepuncture on site. For the remaining 4 participants, HCV RNA point of care testing was completed by using the Xpert® HCV Viral Load Fingerstick test (Cepheid, California, USA), with assistance from Nurses on Wheels, a Franciscan Aid organization. A financial incentive of 200 NOK was offered and accepted by 8 individuals that had travel expenses related to the HCV RNA testing.

1.2 Virologic outcomes and observation time

When assessing virologic outcomes (SVR), all individuals in the ITT analysis were given an observation time. Observation time was defined as time between inclusion date and SVR≥ 4 if SVR≥ 4 had been achieved within 2 years of study enrollment. For individuals that did not achieve SVR≥ 4 within 2 years of enrollment, the observation period was defined as: time between inclusion and date of death for participants that died during the study period, or time between inclusion date and date of a positive HCV RNA sample (implying reinfection, relapse, or virologic failure). For individuals that did not reach SVR4 within 2 years of enrollment, died, or experienced an event with an HCV RNA positive sample, observation time was defined as 2 years, starting at study inclusion.

1.3 SVR rates for participants experiencing reinfection with spontaneous clearance

All 4 individuals experiencing reinfection achieved the primary endpoint. However, 2 of these individuals experienced spontaneous clearance (after reinfection), and undetectable HCV RNA was not a consequence of DAA treatment. The authors have chosen to report these cases as SVR, as DAA treatment had been administered for the primary HCV infection with virologic response (SVR 2-4).

1.4 WGS analysis and genotyping

Recurrent HCV RNA positive follow-up samples were stored at -70^0 C, before being shipped to the NIPH for whole genome sequencing (WGS). WGS was performed using the KAPA Hyper Prep kit (Roche, Indianapolis, IN, US) for library preparation and the VirCapSeq-VERT probes (Roche) for enrichment before pooling and sequencing on the Illumina MiSeq-platform. Sequence data was first quality checked and trimmed, followed by an initial reference-based mapping against a reference database, including all currently recognized genotypes and sub-genotypes. A consecutive reference-based mapping and whole genome assembly followed, verifying the correct sub-genotype. Resistance-

associated substitutions was determined in NS3, NS5, and NS5B by using the HCV-GLUE platform. A minimum HCV RNA quantity of 10 000 IU/ml was used initially but changed to 50.000IU/ml during the study as the first threshold resulted in too many incomplete results. Regarding genotyping, a collaborating microbiological department had in some cases completed genotyping using the Versant HCV Genotype 2.0-line probe assay (Simens Healthineers; Germany) prior to shipment of the samples to the NIPH for WGS analysis.

2. Supplementary results

HCV RNA samples collected at the end of follow-up after the 2-year time frame, SVR≥ 4 was accomplished by 63 of 98 (64.2% [95% CI 54.0-73.7]) during intervention conditions and by 72 of 102 (71% [95% CI 61-79]) during control conditions (risk difference -6% [95% CI -19-7]).

3. Supplementary Table S1.

Baseline characteristics for the ITT population, summarized by total, SVR≥ 4 within 2 years of inclusion, and SVR≥ 4 for total observation time. Numbers are shown as n (%) unless otherwise indicated. Missing values are excluded from percentages. SD = standard deviation;

		SVR≥4 within 2 years		SVR≥4 for total observation time	
**Variable**	**Total (n=200)**	**Yes (n=116)**	**No (n=84)**	**Yes (n=135)**	**No (n=65)**
**Age, mean (SD)**	47.4 (13)	46.5 (12)	48.6 (14)	46.4 (12)	49.6 (14)
**Age groups**					
20-49	112 (56)	69 (60)	43 (51)	80 (59)	32 (49)
50-80	88 (44)	47 (40)	41 (49)	55 (41)	33 (51)
**Sex**					
Male	145 (73)	81 (70)	64 (76)	95 (73)	50 (73)
Female	55 (28)	35 (30)	20 (24)	36 (27)	19 (27)
**Housing status**					
Stable	124 (62)	67 (58)	57 (68)	78 (58)	46 (71)
Unstable	76 (38)	49 (42)	27 (32)	57 (42)	19 (29)
**Recent (past 3 months) injecting drug use**					
Yes	121 (61)	75 (65)	46 (56)	85 (65)	36 (52)
No	79 (40)	41 (35)	38 (45)	46 (35)	33 (48)
**Preferred injected drug†**					
Heroin	114 (64)	66 (66)	48 (62)	79 (66)	35 (60)
Amphetamines	51 (29)	26 (26)	25 (33)	31 (26)	20 (35)
Other/mixed	12 (7)	8 (8)	4 (5)	9 (8)	3 (5)
**Current opioid agonist therapy**					
Yes	90 (45)	56 (48)	34 (41)	65 (48)	25 (39)
No	110 (55)	60 (52)	50 (59)	70 (52)	40 (61)
**Liver cirrhosis¶**					
Mild or no liver fibrosis	156 (80)	93 (81)	63 (78)	110 (82)	46 (74)
Liver cirrhosis	40 (20)	22 (19)	18 (22)	24 (18)	16 (26)
**Charlson comorbidity index**					

0-1	101 (51)	63 (54)	38 (45)	74 (55)	27 (42)
2-3	43 (21)	26 (23)	17 (20)	30 (22)	13 (20)
≥ 4	56 (28)	27 (23)	29 (35)	31 (23)	25 (38)
**Discipline**					
Internal medicine	107 (53)	53 (46)	54 (64)	64 (47)	43 (66)
Addiction	65 (33)	43 (37)	22 (26)	51 (38)	14 (22)
Psychiatry	28 (14)	20 (17)	8 (10)	20 (15)	8 (12)

† Among those with a history of injecting drug use; missing data for 7 participants (2 intervention, 5 control)

¶ Based on liver stiffness measurements in 86, FIB-4 (fibrosis-4) index in 107, and imaging in 3 participants: missing data for 4 control participants

4. Supplementary Table S2

Intention to treat SVR≥ 4 within 2 years of enrolment vs. SVR≥ 4 for total observation time in subgroups according to intervention and control conditions.

	SVR≥4 within 2 years		SVR≥4 for the total observation time	
**Variable**	**Intervention (n=98)**	**Control (n=102)**	**Intervention (n=98)**	**Control (n=102)**
	**n/N (%)**	**n/N (%)**	**n/N (%)**	**n/N (%)**
**Total**	59/98 (60)	57/102 (56)	63/98 (64)	72/102 (71)
**Age groups**				
20-34	10/20 (50)	13/19 (68)	11/20 (55)	17/19 (90)
35-49	19/28 (68)	27/45 (60)	19/28 (68)	33/45 (73)
50-80	30/50 (60)	17/38 (45)	33/50 (66)	22/38 (58)
**Sex**				
Male	39/69 (57)	42/76 (55)	43/69 (62)	54/76 (71)
Female	20/29 (69)	15/26 (58)	20/29 (70)	18/26 (69)
**Housing status**				
Stable	32/64 50)	35/60 (58)	35/64 (55)	43/60 (72)
Unstable	27/34 (79)	22/42 (52)	28/34 (82)	29/42 (69)
**Recent (past 3 months) injecting drug use**				
Yes	37/58 (64)	38/63 (60)	39/58 (67)	48/63 (76)
No	22/40 (55)	19/39 (49)	24/40 (60)	24/39 (62)
**Preferred injected drug†**				
Heroin	32/54 (59)	34/60 (64)	35/54 (65)	44/60 (73)
Amphetamines	14/25 (56)	12/26 (46)	15/25 (60)	16/26 (62)
Other/mixed	4/6 (67)	4/6 (67)	4/6 (67)	5/6 (83)
**Current opioid agonist therapy**				
Yes	27/38 (74)	29/52 (56)	29/38 (76)	36/52 (70)
No	32/60 (53)	28/50 (56)	34/60 (57)	36/50 (72)
**Liver cirrhosis¶**				
Mild or no liver fibrosis	46/76 (61)	47/80 (59)	49/76 (65)	61/80 (76)
Liver cirrhosis	13/22 (59)	9/18 (50)	14/22 (64)	10/18 (56)

**Charlson comorbidity index**				
0-1	26/41 (63)	37/60 (62)	27/41 (66)	47/60 (78)
2-3	20/30 (67)	6/13 (46)	22/30 (73)	8/13 (62)
≥ 4	13/27 (48)	14/29 (48)	14/27 (52)	17/29 (59)
**Discipline**				
Internal medicine	34/57 (60)	19/50 (38)	37/57 (65)	27/50 (54)
Addiction	16/25 (64)	27/40 (68)	17/25 (68)	34/40 (85)
Psychiatry	9/16 (56)	11/12 (92)	9/16 (56)	11/12 (92)

† Among those with a history of injecting drug use; missing data for 7 participants (2 intervention, 5 control)
¶ Based on liver stiffness measurements in 86, FIB-4 (fibrosis-4) index in 107, and imaging in 3 participants: missing data for 4 control participants

5. Supplementary Table S3.

Virologic outcomes and reasons for failure to accomplish SVR≥ 4 within 2 years of enrollment according to intervention and controls conditions.

	Total (n=200)	Intervention (n=98)	Control (n=102)
**SVR≥ 4**	116 (58)	59 (60)	57 (56)
**Failure to achieve SVR≥ 4**			
ETR with missing SVR data	5 (3)	4 (4)	1 (1)
Missing SVR data	18 (9)	16 (16)	2 (2)
SVR outside time limit	19 (9)	4 (4)	15 (15)
Treatment failure	5 (3)	4 (4)	1 (1)
Untreated	37 (19)	11 (12)	26 (25)

Numbers are shown as n (%). SVR, sustained virologic response; ETR, end of treatment response

6. Supplementary Table S4.
Subgroup incidence rates of reinfection, summarized by total, intervention, and control conditions. Missing values are excluded from percentages.

	**Total (n=145)**		**Intervention (n=71)**		**Control (n=74)**	
**Variable**	Events, n/N (%)	Incidence rate, per 100 PY (95% CI)	Events (n %)	Incidence rate, per 100 PY (95% CI)	Events (n %)	Incidence rate per 100 PY (95% CI)
**Overall**	4/145 (3)	4 (1-10)	1/71 (1)	2 (0-14)	3/74 (4)	5 (1-14)
**Age groups**						
20-49	4/84 (5)	6 (2-16)	1/33 (3)	5 (0-27)	3/51 (6)	7 (1-20)
50-80	0/61 (0)	0 (0-9)	0/38 (0)	0 (0-18)	0/23 (0)	0 (0-17)
**Sex**						
Male	4/106 (4)	5 (1-13)	1/50 (2)	3 (0-18)	3/56 (5)	6 (1-18)
Female	0/39 (0)	0 (0-14)	0/21 (0)	0 (0-32)	0/18 (0)	0 (0-24)
**Housing status**						
Stable	2/85 (2)	3 (0-12)	1/42 (2)	4 (0-23)	1/43 (2)	3 (0-14)
Unstable	2/60 (3)	4 (1-17)	0/29 (0)	0 (0-21)	2/31 (7)	8 (1-28)
**Recent injecting drug use**						
Yes	3/90 (3)	4 (1-12)	1/41 (2)	3 (0-21)	2/49 (4)	4 (1-16)
No	1/55 (2)	3 (0-17)	0/30 (0)	0 (0-27)	1/25 (4)	5 (0-30)
**Preferred injected drug†**						
Heroin	3/83 (4)	5 (0-14)	1/38 (3)	3 (0-22)	2/45 (4)	5 (0-19)
Amphetamines	0/36 (0)	0 (0-13)	0/19 (0)	0 (0-37)	0/17 (0)	0 (0-21)
Other/mixed	1/9 (11)	13 (0-72)	0/4 (0)	0 (0-177) *	1/5 (20)	18 (0-98)
**Current opioid agonist therapy**						
Yes	3/68 (4)	6 (1-17)	0/31 (0)	0 (0-19)	3/37 (8)	9 (2-26)
No	1/77 (1)	2 (0-11)	1/40 (3)	4 (0-25)	0/37 (0)	0 (0-12)
**Liver cirrhosis¶**						
Mild or no liver fibrosis	4/118 (3)	4 (1-11)	1/55 (2)	3 (0-17)	3/63 (5)	5 (1-15)
Liver cirrhosis	0/26 (0)	0 (0-27)	0/16 (0)	0 (0-44)	0/10 (0)	0 (0-68)
**Charlson comorbidity index**						
0-1	4/76 (5)	7 (2-17)	1/28 (4)	5 (0-29)	3/48 (6)	7 (2-21)
2-3	0/34 (0)	0 (0-16) *	0/25 (0)	0 (0-26) *	0/9 (0)	0 (0-40) *
≥ 4	0/35 (0)	0 (0-17) *	0/18 (0)	0 (0-47) *	0/17 (0)	0 (0-25) *

**Discipline**						
Internal medicine	0/70 (0)	0 (0-7) *	0/42 (0)	0 (0-14) *	0/28 (0)	0 (0-14) *
Addiction	4/53 (8)	10 (3-25)	1/18 (6)	10 (0-54)	3/35 (9)	10 (2-29)
Psychiatry	0/22 (0)	0 (0-28) *	0/11 (0)	0 (0-83) *	0/11 (0)	0 (0-43) *

† Among those with a history of injecting drug use; missing data for 3 participants (1 intervention, 2 control)

¶ Based on liver stiffness measurements in 67, FIB-4 (fibrosis-4) index in 77: missing data for 1 control participant

* One sided 97.5% confidence interval

7. Supplementary Figure S1.

Kaplan-Meier estimates of time to reinfection according to (A) sex, (B) age groups, (C) housing status, and (D) intervention. Incidence rates with 95% confidence intervals are shown.

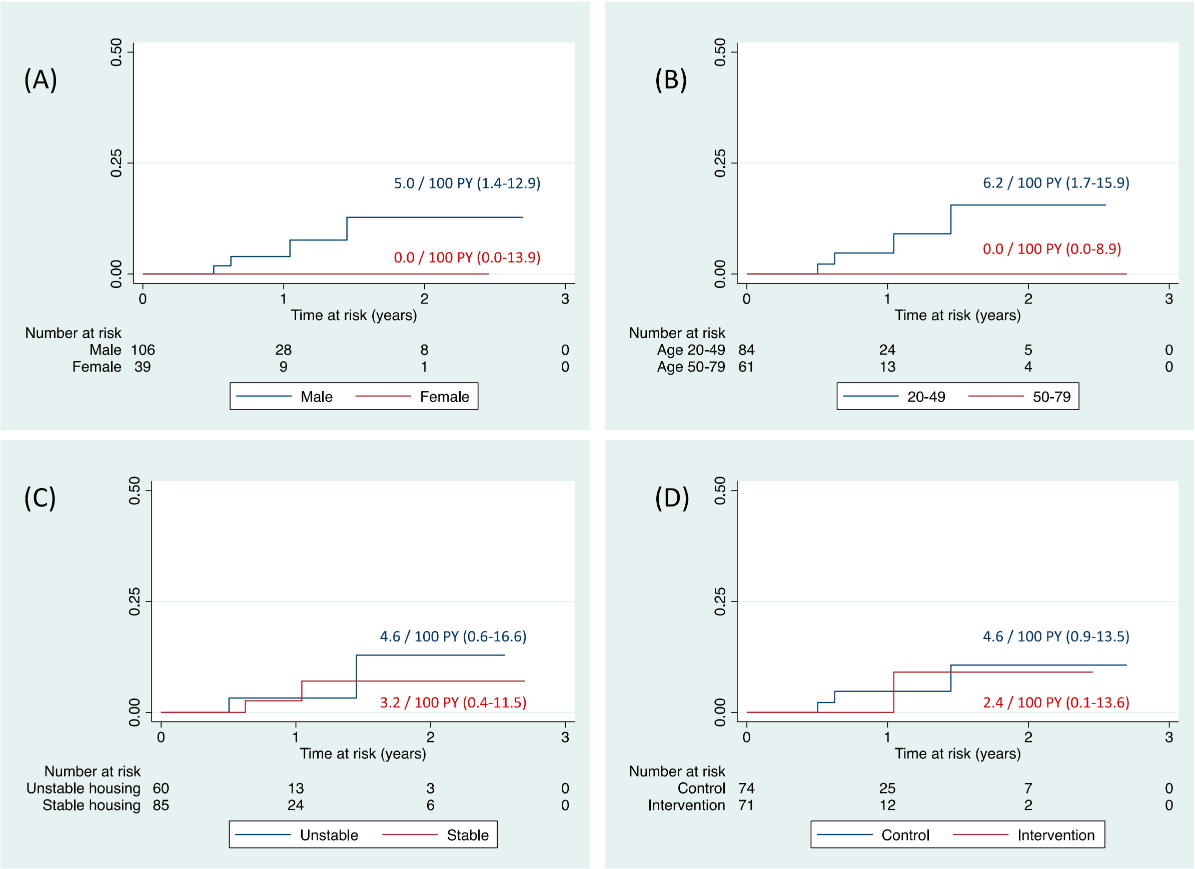

Supplement: Supplementary file 1 [file viruses-16-00858-s001.zip › viruses-2973968-supplementary.pdf]
